# Trafficking of neuronal calcium channels

**DOI:** 10.1042/NS20160003

**Published:** 2017-02-20

**Authors:** Norbert Weiss, Gerald W. Zamponi

**Affiliations:** 1 Institute of Organic Chemistry and Biochemistry, Czech Academy of Sciences, Prague, Czech Republic; 2 Department of Physiology and Pharmacology, Hotchkiss Brain Institute and Alberta Children's Hospital Research Institute, Cumming School of Medicine, University of Calgary, Calgary, Canada

**Keywords:** ancillary subunit, calcium channels, glycosylation, Stac adaptor proteins, trafficking, ubiquitination, voltage-gated calcium channels

## Abstract

Neuronal voltage-gated calcium channels (VGCCs) serve complex yet essential physiological functions via their pivotal role in translating electrical signals into intracellular calcium elevations and associated downstream signalling pathways. There are a number of regulatory mechanisms to ensure a dynamic control of the number of channels embedded in the plasma membrane, whereas alteration of the surface expression of VGCCs has been linked to various disease conditions. Here, we provide an overview of the mechanisms that control the trafficking of VGCCs to and from the plasma membrane, and discuss their implication in pathophysiological conditions and their potential as therapeutic targets.

## Introduction

**Figure 1:**
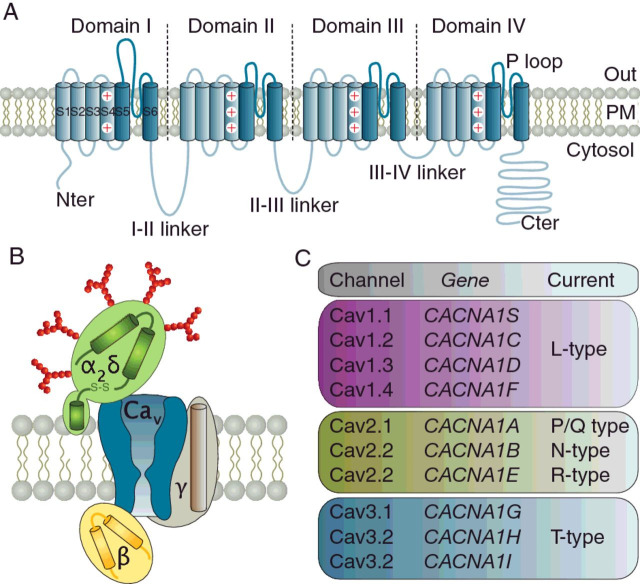
Molecular composition and diversity of VGCCs (**A**) Membrane topology of the Ca_v_α_1-_pore forming subunit. It consists of four repeats (domain I to IV) made out of six transmembrane segments (S1 to S6) and connected by cytosolic regions (linker I-II to III-IV). The arginine-rich S4 segments of each of the four domains form the voltage sensor, whereas the pore forming loops connecting segments S5 and S6 comprise the Ca^2+^ selectivity elements of the channel. (**B**) Schematic representation of the VGCC complex, consisting of the Ca_v_-pore forming subunit surrounded by β (yellow), α_2_δ (green) and γ (brown) ancillary subunits. (**C**) Diversity of VGCCs. The HVA subfamily is composed of Ca_v_1.x (L-type) and Ca_v_2.x (P/Q-, N- and R-type) channels, whereas Ca_v_3.x channels (T-type) form the LVA subfamily.

Within neurons, calcium ions (Ca^2+^) are essential for regulating a myriad of cellular processes and ultimately physiological functions [1]. Like other cells, neurons use both extracellular and intracellular sources of Ca^2+^, and much has been learned about the mechanisms and players responsible for mobilizing Ca^2+^ [2]. Voltage-gated calcium channels (VGCCs) are essential for the initiation of the Ca^2+^ signalling cascades that are triggered by membrane depolarizations. They play a pivotal role in translating surface electrical signals into Ca^2+^ influx and intracellular Ca^2+^ elevations. These in turn support a number of cellular mechanisms ranging from synaptic integration to Ca^2+^-evoked gene transcription and neurotransmitter release [3,4]. VGCCs are pore-forming macromolecular complexes embedded in the plasma membrane of all kind of nerve cells. To date, ten genes encode for the pore-forming Ca_v_α_1_ subunit of mammalian VGCCs and fall into two subfamilies: seven genes encode the high-voltage-activated (HVA) channels and consist of L-type (Ca_v_1.1 to Ca_v_1.4), P/Q-type (Ca_v_2.1), N-type (Ca_v_2.2), R-type (Ca_v_2.3) and three genes encode the low-voltage-activated (LVA) channels represented exclusively by T-type channels (Ca_v_3.1 to Ca_v_3.3) (Figure 1) [4–6]. Ca_v_α_1_ consists of four homologous membrane domains, each of them containing six transmembrane helices (S1–S6) and an extracellular re-entrant loop (so-called P-loop) responsible for shaping the pore and the selectivity filter of the Ca^2+^ channel. The four membrane domains are connected via variable cytoplasmic (loops I-II, II-III, III-IV) and N- and C-terminal regions that form molecular hubs for binding of regulatory proteins and post-translational modification of the channel. In addition to the Ca_v_-pore forming subunit, HVA channel complexes contain a number of ancillary subunits, namely β, α_2_δ, and in some circumstances γ [4]. Initially identified with the skeletal dihydropyridine receptor complex [7], they are widely expressed in the nervous system where they associated with most of the HVA channel isoforms and are essential for cell surface expression and functioning of the channel [8]. In contrast, these ancillary subunits have little, if any, influence on the expression and function of T-type channels [9–11].

A number of regulatory mechanisms act on VGCCs to indirectly regulate the amplitude, duration and subcellular localization of the Ca^2+^ signal. Indeed, control of VGCCs occurs at many different levels, starting with the control of channel subunits at the mRNA level. The modulation of VGCCs by RNA editing and alternative pre-mRNA splicing has been previously reviewed [12] and is considered a very important mechanism for diversifying calcium signalling. In addition, much attention has focused on the mechanisms that regulate the gating of the channel at the cell surface, including but are not limited to the modulation by synaptic proteins [13]; channel dimerization with other ion channels and receptors [14]; and the activation of G-protein-coupled receptors and downstream signalling pathways including phosphorylation [15]. Control of VGCCs may also occur at the level of the channel subunit trafficking to and from the plasma membrane [16]. Although the mechanisms controlling the expression of VGCCs along with those controlling their subcellular targeting are by far the least understood, a number of recent studies have uncovered essential factors and signalling pathways to ensure an optimal density of channels in the plasma membrane. In this review, we consider mechanisms that control the trafficking of VGCCs. We review the role of channel ancillary subunits and other interacting proteins in the expression of the channel. We also consider the importance of post-translational modifications including glycosylation and ubiquitination in the trafficking and stability of the channel at the cell surface. Finally, we discuss the implication of these regulatory mechanisms in chronic disorders of VGCCs, and analyse their potential as therapeutic targets.

## Ancillary subunit-dependent trafficking of VGCCs

### β-subunit

The β-subunit of VGCCs is a 55-kDa cytosolic protein that belongs to the membrane-associated guanylate kinase (GK) family. It consists of SH3 and GK domains that are highly conserved among the different β isoforms (β_1_ to β_4_), and linked by a variable HOOK region [17–19]. The β-subunit binds with high affinity (*in vitro K*_d_ of approximately 5 nM) to a conserved motif (QQ-E–L-GY–WI–E) so called alpha-interaction domain (AID) within the cytoplasmic linker between repeats I and II of the Ca_v_α_1_ subunits of HVA channels [20]. In addition, crystal structures of different AID–β-core complexes have defined the counterpart biding region within the β-subunit called AID-binding pocket (ABP) formed by a hydrophobic groove in the GK domain [21–23]. The first evidence for a role of the β-subunit in the expression of VGCCs arose from co-expression studies in *Xenopus* oocytes and mammalian cells where a spectrum of effects were observed including an increase in the current amplitude by orders of magnitude [24–26]. In addition, knockdown of β-subunits in dorsal root ganglion (DRG) neurons using a pan oligonucleotide antisense against all β-subunit isoforms resulted in a significant, albeit incomplete, decrease in the calcium current [27]. It is interesting to note that in contrast with the effects that have been reported in heterologous expression systems, Ca_v_1.2 current densities in cardiomyocytes lacking the Ca_v_β_2_ subunit show only a moderate reduction in Ca_v_1.2 channel expression levels [28]. There appeared to be no compensation from other Ca_v_β-subunit isoforms, but it is possible that other compensatory mechanisms that increase Ca_v_1.2 channel trafficking may have been activated.

**Figure 2:**
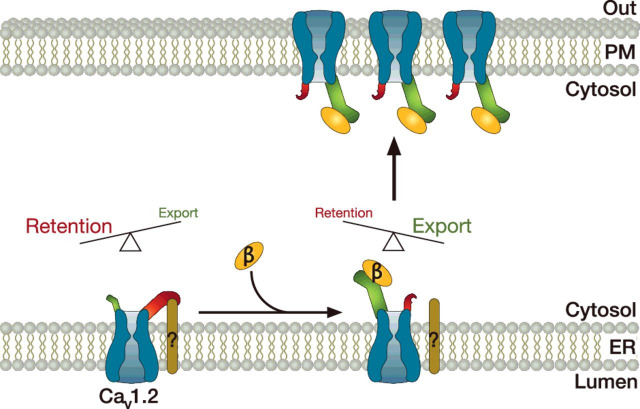
Control of Ca_v_1.2 channel trafficking by β-subunits The Ca_v_1.2 channel contains an ER ‘export’ signal (green) located in the I-II linker, and an ER retention signal (red) made of various molecular determinants located within the II-III and III-IV linkers and the N- and C-terminal regions of the channel, which possibly interact with a yet unknown ER resident protein. In the absence of β-subunit, the retention signal is stronger than the ‘export’ signal and the channel is retained in the ER. Binding of a β-subunit on to the I-II linker triggers a molecular reorganization of the intracellular regions of the channel that diminishes the strength of the retention signal relative to the ‘export’ signal, leading to the exit of the channel from the ER and its trafficking to the cell surface.

Although β-subunit-dependent potentiation of the Ca^2+^ current is multifactorial and clearly includes an increase in the opening probability of the channel [29–33], a role in the trafficking of the channel to the cell surface rapidly emerged as the key function of these subunits. Based on the observation that a CD8 fusion construct of the I-II linker of Ca_v_2.1 is retained in the endoplasmic reticulum (ER) unless co-expressed with a β-subunit prompted, it was proposed that the β-subunit may shield an ER retention signal in the I-II linker of the Ca_v_α_1_ subunit, thus enhancing the exit of the channel from the ER [34]. However, this hypothesis was later challenged by a number of additional studies. First, an ER retention signal was never identified in the AID and surrounding regions, and deletion of the AID domain in Ca_v_2.1 failed to mimic the effect of the β-subunit in terms of surface expression of the channel [17]. Second, the I-II linkers from various Ca_v_α_1_ isoforms, despite all containing the AID motif, do not cause ER retention of CD8 or CD4 fusion proteins [35,36]. Third, substitution of the I-II linker of the LVA Ca_v_3.1 channel (which does not require the presence of a β-subunit to translocate to the cell surface) with the domain I-II linker of different HVA channels including Ca_v_1.2, Ca_v_2.1 and Ca_v_2.2 did not produce ER retention of the T-type channel but in contrast caused an up-regulation of the current [37,38]. In order to re-evaluate the mechanisms of β-mediated up-regulation of HVA channels, all of the T-type Ca_v_3.1 channel intracellular linkers were systematically swapped with corresponding regions of the Ca_v_1.2 channel, individually or in combination, and surface expression of the chimaeric channel was examined in the presence or absence of a β-subunit [38]. This elegant study suggested that the I-II linker of Ca_v_1.2 in fact contains an ER ‘export’ signal, whereas all the other intracellular regions were found to contain some elements of ER retention signals. In an attempt to reconcile these divergent results, it was proposed that binding of the β-subunit to the I-II loop of the Ca_v_α_1_ subunit may restructure the intramolecular arrangement of the intracellular linkers, favouring the ‘export’ conformation over the retention signal (Figure 2) [38]. A number of additional studies also reported a cross-talk between β-subunit and proteasome-dependent degradation of the channel. This aspect is further discussed in the section concerning the control of VGCC expression by the ubiquitin proteasome system.

Altogether, these findings indicate that the β-subunit plays an essential role in the expression of HVA channels at the cell surface by stimulating the exit of the channel from the ER, and also by diverting the channel from the proteasomal degradation pathway. Whether these two mechanisms are independent or linked with each other needs to be further analysed. On the other hand, it is likely that a pool of channels may remain stagnant in the ER, protected from degradation, and awaiting for a trigger signal to translocate to the cell surface to rapidly support cell adaptation to various stimuli or environmental cellular conditions.

### α_2_δ subunit

The α_2_δ subunit is an extracellular component of the channel complex, which is essential for cell surface trafficking, functional expression and pharmacological modulation of the Ca_v_-pore forming subunit [39–41]. Each of the four α_2_δ isoforms (α_2_δ-1 to α_2_δ-4) is transcribed and translated from a single gene, and undergoes a number of post-translational modifications that have important consequences on the fate and behaviour of the protein. First, proteolytic cleavage generates α_2_ and δ peptides that remain associated by disulfide bonds. Second, a glycosylphosphatidylinositol (GPI) moiety is attached to the C-terminal region of the δ peptide, and contributes to the anchoring of the protein to the plasma membrane after it has been secreted [42]. Third, α_2_δ is heavily glycosylated, which accounts for approximately a third of its total molecular weight [43]. The role of glycosylation of α_2_δ in the trafficking of HVA channels is discussed later in this review. All α_2_δ subunits contain a Von Willebrand factor A (VWF-A or VWA) domain, which represents a dinucleotide binding fold with a metal ion adhesion (MIDAS) motif and participates in a number of divalent cation interactions [44]. This subunit also contains two bacterial chemosensory-like domains (Cache) located downstream of the VWA domain [45]. The α_2_δ subunits are present in both skeletal [7], cardiac [46] and neuronal channel complexes [47,48], and it is likely that all HVA channels can associate with α_2_δ. Similar to what has been shown with the β-subunit, α_2_δ subunits increase surface expression of various Ca_v_α_1_ isoforms including Ca_v_1.2 [31,49–51], Ca_v_2.1 [51–55], Ca_v_2.2 [50,51,54,56], both in co-expression studies and native conditions. In contrast, α_2_δ seems to be less essential for the expression of Ca_v_2.3 at the cell surface [30,57]. In addition to the effects of this subunit on channel trafficking, an increased opening probability of the channel in the presence of α_2_δ has been reported [29], and proteolytic cleavage of α_2_δ is essential for voltage-dependent activation of the channel at the cell surface [58]. Moreover, decreased internalization of the channels from the cell surface in the presence of α_2_δ subunits has been reported [59].

The exact mechanism by which α_2_δ potentiates surface expression of HVA channels is still up for debate. It was reported that the MIDAS motif in the VWA domain of α_2_δ-1 and α_2_δ-2 is critical to mediate this process [54,60]. Considering that the VWA domain is involved in protein–protein interactions, it is a possibility that α_2_δ may interact with proteins involved in the trafficking of the channel, or directly with the Ca_v_ subunit to promote the trafficking of the channel complex. Consistent with this notion, the recent structure of the skeletal muscle Ca_v_1.1 channel complex determined by single-particle cryo-EM shed light on the molecular determinants involved in α_2_δ/Ca_v_α_1_ interactions [61]. Consistent with previous biochemical studies [62], α_2_δ interacts with the extracellular loops of repeats I to III of the Ca_v_α_1_ subunit via its VWA and Cache1 domains. In addition, there is pharmacological evidence for a role of α_2_δ/Ca_v_α_1_ interaction in the trafficking of the channel. For instance, the antiepileptic/antiallodynic drugs gabapentin and pregabalin [63–65] target α_2_δ [66,67]. Although the exact binding determinants of gabapentinoid drugs have not all been identified yet, a number of amino acids, especially the third arginine (R) in the RRR motif located upstream the VWA domain, were shown to contribute to the binding [68,69]. Interestingly, gabapentin was found to not only preclude the trafficking of Ca_v_2.1 and Ca_v_2.2 channels to the cell surface [56], but also counteract the increased expression of Ca_v_2.2 channels associated with chronic pain conditions [70]. In addition, gabapentin inhibits rab11-dependent recycling of α_2_δ-2 from post-Golgi compartments to the cell surface, which may in turn reduce cell surface expression of the channel complex [71]. Interestingly, the mutation of the third arginine in the RRR motif to an alanine (RRA) that alters the binding of gabapentin was shown to abolish the ability of α_2_δ to enhance surface expression of the channel [56,72], suggesting that α_2_δ-dependent expression of the channel relies on the coupling with the Ca_v_α_1_ subunit, and that gabapentinoid drugs exert their effect by disrupting the Ca_v_α_1_/α_2_δ complex.

Altogether, it is unambiguous that α_2_δ is essential for the trafficking and surface expression of HVA channels. However, it is also apparent that the exact mechanisms by which α_2_δ potentiates the trafficking of the channel remain elusive. Although a biochemical interaction between α_2_δ and the Ca_v_α_1_ subunit seems to be required, it is still elusive the way Ca_v_α_1_/α_2_δ complex traffics to the cell surface and whether ER retention signals are involved in this regulation.

### γ-subunit

The γ-subunit is a 33-kDa transmembrane protein initially described as a component of the skeletal muscle channel complex [7], and it was later demonstrated that nerve cells also express various homologues of the γ-subunit. To date, eight isoforms have been documented (γ_1_ to γ_8_), but compared with β and α_2_δ subunits, little attention has been focused on the γ-subunit, probably because of its relative mild effect on the channel function when expressed in *Xenopus* oocytes or mammalian cells [73–78]. However, evidence exists for a role of γ in the modulation of native channels. For instance, the role of γ_2_, also known as stargazin [79], in the modulation of ion channels is best exemplified by its implication in absence seizures in the stargazer mouse that carries a transposon insertion in the gene encoding for γ_2_ [79]. This insertion severely reduces the expression of the γ_2_ subunit [80–82], and is accompanied by an increased current density of both HVA and LVA channels in thalamocortical relay nuclei [83]. However, *in vitro* studies tend to suggest a direct modulation of the channel activity rather than an altered trafficking of the channel *per se*. Indeed, reduced Ca_v_2.2 current density in cells co-expressing γ_7_ is not accompanied by a reduction in the surface expression of the channel protein [84]. Similarly, Ca_v_3.1 T-type currents are inhibited in an atrial cell line expressing γ_6_ but this effect does not appear to be mediated by reduced channel trafficking [85–87]. In contrast, altered trafficking of AMPA receptors from the Golgi apparatus to the plasma membrane was documented in the stargazer mouse, indicating that γ_2_ is essential for surface expression and postsynaptic targeting of the receptor [88,89]. Interestingly, the γ_4_ isoform that shares a high degree of amino acid conservation with γ_2_ was found to be even more effective in trafficking AMPA receptors to the cell surface [90]. Indeed, neuronal γ subunits may not have a primary role as bona fide calcium channel subunits, but are instead rather known to be important constituents of AMPA and kainate receptors. Along these lines, γ_7_ has been shown to exhibit binding to mRNA [91], and it is possible that the effects of co-expression of these subunits with various calcium channel isoforms are more diffusely related to changes in gene expression of channels or their regulatory elements, which in turn may account for altered ion channel trafficking or gating. This may fit with the observation that the related γ_2_ subunit regulates N-type channels indirectly via a G-protein dependent pathway [92].

## Trafficking regulation of VGCCs by other interacting proteins

### Calmodulin

Calmodulin (CaM) is a multifunctional intermediate Ca^2+^-binding protein essential for neuronal signalling [93]. The fact that CaM associates with virtually all HVA channels has led the proposal that this protein might be considered as an integral component of the Ca^2+^ channel complex *per se* [93], and prompted some authors to propose the term ‘calmodulation’ to cover any kind of CaM-dependent regulation of ion channels. The multifaceted regulation of VGCCs by CaM has extensively been reviewed over the past 2 years [94–96]. In this section, we focus exclusively on its specific role in the trafficking of the channel complex. Although a number of domains located in the C-terminal region of the Ca_v_α_1_ subunit of HVA channels have been shown to be involved in the binding of Ca^2+^/CaM with the channel, the IQ domain has emerged as prerequisite for the binding of Ca^2+^ free Apo-CaM. Although the role of CaM in the modulation of channel activity has been extensively documented, its role in the trafficking of the channel has remained controversial. Initial data suggested that CaM has no effect on the surface expression of Ca_v_1.2 in HEK-293 cells, either in the presence or absence of the ancillary subunits β and α_2_δ [97]. In contrast, surface expression of Ca_v_1.2 channels lacking the PreIQ3 region located upstream of the IQ domain and important for the binding of Ca^2+^/CaM is completely abolished despite the presence of the β_2a_ subunit [98]. Consistent with a role of CaM in the expression of VGCCs, trafficking of Ca_v_1.2 channels to distal dendrites of hippocampal neurons is accelerated by Ca^2+^/CaM but not by Apo-CaM [99]. In addition, a number of studies have reported that mutations in the EF-hand domain of Ca_v_1.2 channels alter current amplitude [100–102]. Although these mutations certainly altered the gating modulation of the channel [99,103], it is also a possibility that CaM-dependent trafficking of the channel was hampered. In addition to the CaM-binding domains in the C-terminal region of the channel, the N-terminal region of Ca_v_1.2 contains two additional biding regions that are not present in Ca_v_2.x channels [104–106]. However, there is no obvious evidence for a role in the trafficking of the channel. Recently, a CaM kinase II-biding site was identified in the N-terminal region of Ca_v_1.2 important for cell surface expression of the channel [107]. Interestingly, disruption of the CaMKII-binding site caused a significant reduction in the expression of the channel protein at the cell surface when expressed in tsA-201 cells. Whether a cross-talk exists between the binding of Ca^2+^/CaM and CaMKII remains to be analysed.

We note that many studies that investigate the role of CaM and/or CamKII interactions rely on point mutations in the critical binding domains on the channel. In interpreting the functional data obtained with mutant constructs, it is thus important to consider the possibility that mutations may have more global effects on channel folding and trafficking *per se*. Nonetheless, these data shed light on a potential role for Ca^2+^/CaM in regulating cell surface expression of VGCCs, even though the underlying mechanisms supporting this regulation remain to be precisely elucidated.

### Stac adaptor proteins

Stac adaptor proteins form a family of SH3 and cysteine-rich domain-containing proteins whose primary functions have remained largely unknown [108]. Although Stac3 is essentially expressed in skeletal muscle, Stac1 and Stac2 are widely expressed in the peripheral and central nervous system [109]. Initial studies reported an essential role for Stac3 in the assembly and functioning of the excitation–contraction coupling machinery and maturation of skeletal muscle [110–113]. A role for Stac3 in the trafficking of Ca_v_1.1 channels to the cell surface was then documented in tsA-201 cells [114,115]. These results prompted us to analyse whether Stac proteins may also contribute to the expression of neuronal VGCCs, especially T-type channels. As already mentioned, surface expression of LVA channels is barely influenced by the usual channel ancillary subunits, and although the I-II loop was reported to contribute to the expression of the channel [116,117], little is known about the molecular mechanisms controlling the trafficking of T-type channels. Our laboratory has shown that Stac1 binds to the distal region of the N-terminus of Ca_v_3.2 channels and potentiates T-type currents in tsA-201 cells [118]. Although the main gating properties of the channel were not affected by Stac1, surface expression of Ca_v_3.2 was increased suggesting that the enhanced T-type conductance was essentially caused by an increased expression of the channel at the plasma membrane. Whether Stac1 potentiates the trafficking of Ca_v_3.2 to the cell surface or stabilizes the channel in the plasma membrane remains to be analysed [119].

Altogether, Stac proteins are emerging as important modulators of T-type channel trafficking, and likely have a broader implication in the expression of VGCCs in general.

### Kelch-like 1

**Figure 3:**
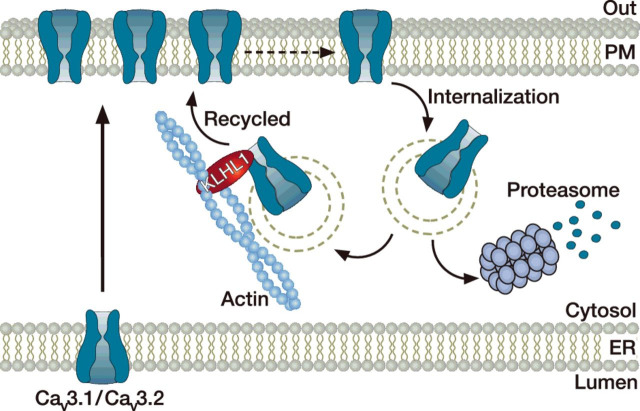
KLHL1-dependent trafficking of T-type channels The steady-state expression of T-type channels at the cell surface results from the number of channels coming to and getting internalized from the plasma membrane. Although T-type channels are internalized and degraded, expression of the actin-binding protein KLHL1 allows the recycling and re-insertion of the channels into the plasma membrane.

Kelch-like 1 (KLHL1) belongs to a family of actin-organizing proteins that interacts with actin and is mostly expressed in neuronal tissues. The Ca^2+^ conductance in tsA-201 cells expressing Ca_v_2.1, Ca_v_3.1 and Ca_v_3.2 was found increased upon co-expression of KLHL1 [120,121]. This was further analysed for Ca_v_3.1 and Ca_v_3.2 channels and shown to be due to increased surface expression of the channel proteins. Biochemical studies also indicated that KLHL1 can be immunoprecipitated with the channels, but the detailed molecular determinants of the interaction have not been characterized. In addition, although pharmacological disruption of actin filaments did not affect the activity of T-type channels *per se*, it did eliminate KLHL1-dependent potentiation of the channels [120]. Similar observations were made upon disruption of endosomal recycling suggesting that KLHL1 enhances surface expression of T-type channels by potentiating their re-insertion into the plasma membrane from recycling endosomes (Figure 3) [120]. The role of KLHL1 in the expression of VGCCs in further supported by the observation that shRNA knockdown of KLHL1 in hippocampal neurons in culture caused a dramatic decrease in both HVA and LVA Ca^2+^ currents [122]. These results are consistent with the observation that Ca_v_2.1 and Ca_v_3.2 are down-regulated in hippocampal neurons from KLHL1 KO mice [123]. However, concomitant up-regulation of Ca_v_1.2 and Ca_v_3.1 was also observed in KLHL1 KO hippocampal neurons, possibly reflecting the occurrence of a compensatory process. Nevertheless and consistent with the presynaptic role of Ca_v_2.1 and Ca_v_3.2, the synaptic activity of KLHL1 KO neurons remained compromised [123].

Overall, these results emphasize the contribution of KLHL1 and actin-binding proteins in the expression of Ca^2+^ channels at the cell surface, and it remains to be determined if this regulation involves some of the channel molecular determinants previously identified in the trafficking of the channel.

## Role of post-translational modifications in VGCC trafficking

### Ubiquitination and the ubiquitin proteasome system

**Figure 4:**
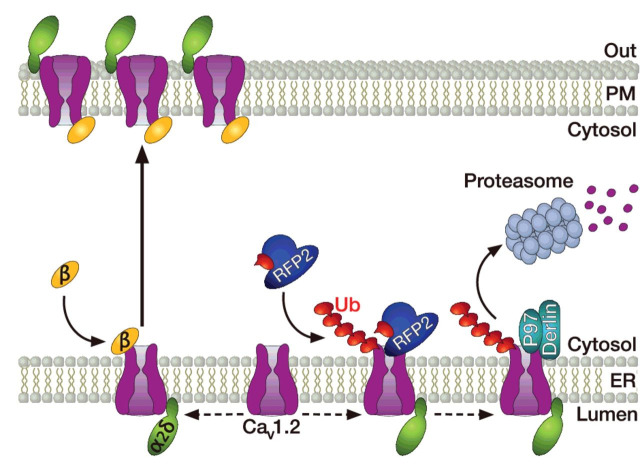
Control of Ca_v_1.2 channel expression by the UPS Ubiquitination (red chain) of Ca_v_1.2 by the ubiquitin ligase RFP2 triggers the association of the channel with ERAD proteins Derlin1 and P97, leading to the degradation of the channel complex by the proteasome system. In the presence of a β-subunit, Ca_v_1.2 is protected from RFP2-dependent ubiquitination, allowing the trafficking of the channel to the cell surface.

**Figure 5:**
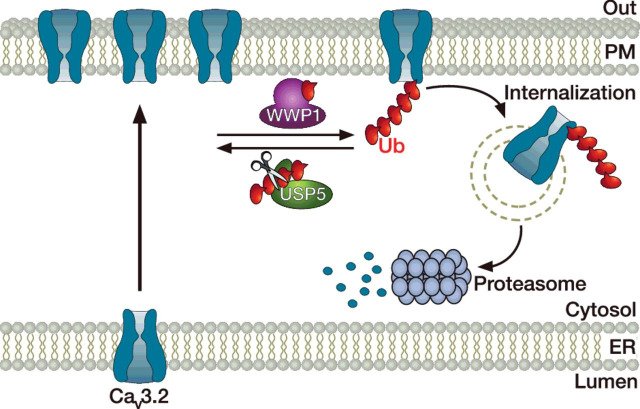
A dynamic ubiquitination/deubiquitination balance controls expression of Ca_v_3.2 channels at the cell surface Although ubiquitination of Ca_v_3.2 by the ubiquitin ligase WWP1 triggers the internalization and degradation of the channel, removal of ubiquitin moieties from the channel protein by the deubiquitinase USP5 increases the stability of the channel in the plasma membrane, leading to enhanced channel surface density.

Ubiquitin-dependent degradation of VGCCs by the ubiquitin-proteasome system (UPS) has emerged as an important means to control the number of channels expressed at the cell surface [124]. Ubiquitination of proteins relies on the attachment of an ubiquitin moiety by an ubiquitin ligase, and dictates the fate of the target protein [125]. Although monoubiquitination generally triggers the internalization of the protein that is either further degraded in lysosomes or recycled back into the plasma membrane, polyubiquitinated proteins are targeted to the proteasome system for proteolysis. Although ubiquitination is a universal mechanism to control the fate of proteins, ubiquitination of VGCCs was only recently documented. For instance, it was proposed that Ca_v_1.2 channels are ubiquitinated by the ubiquitin ligase RFP2, and that ubiquitinated channels interact with key proteins of the endoplasmic reticulum-associated protein degradation (ERAD) complex including derlin-1 and p97, which in turn triggers the targeting of the channel to the proteasome system (Figure 4) [36]. Consistent with this notion, the Ca^2+^ current density and surface expression of Ca_v_1.2 channels was found significantly increased in cells expressing a dominant negative RFP2 construct [36]. In addition, it was demonstrated that the β-subunit prevents RFP2-mediated ubiquitination of Ca_v_1.2, diverting the channel away from its degradation by the proteasome system [36]. This aspect is further supported by the observation that surface expression of the channel in the absence of β-subunit is partially rescued by the proteasome inhibitor MG132. Additional reports indicated that ubiquitination of Ca_v_1.2 channels may also happen via the ubiquitin ligase Nedd4-1 [126], and counteracted by the ubiquitin-specific protease (USP)2-45 [127]. However, despite the fact that USP2-45 can indeed deubiquitinate the channel, Ca^2+^ currents in tsA-201 cells expressing Ca_v_1.2 with USP2-45 were dramatically reduced, suggesting a more complex regulatory mechanism [127]. The ubiquitination of Ca_v_2.2 channels has also been reported, a process that is modulated by the β-subunit as part of its protective function [128,129]. Furthermore, Ca_v_2.2 was shown to interact with a macromolecular complex containing the light chain 1 subunit (LC1) of the microtubule-associated protein 1B (MAP1B) and the ubiquitin conjugase UBE2L3 to control surface expression of the channel [130,131]. Proteasome-dependent degradation of Ca_v_2.2 channels is also enhanced by the binding of the fragile X mental retardation protein (FMRP) with the channel, a process that is defective in fragile X syndrome [132]. In addition, a role for alternative splicing of the pre-mRNA in the modulation of ubiquitin-dependent degradation of the channel has emerged. For instance, the Ca_v_2.2 channels containing exon 37b have increased ubiquitination levels compared with channels containing exon 37a, which is correlated with a reduced expression of the channel [133]. Considering that cell-specific alternative splicing of exon 37a exists in nociceptors, decreased ubiquitination of Ca_v_2.2 e37a may have important consequences in pain processing. This effect might be even amplified by the fact that the adaptor protein complex-1 (AP-1) potentiates the trafficking of Ca_v_2.2 e37a channels from the trans-Golgi to the cell surface, but not of the e37b variant that does not contain the AP-1 binding motif [132]. Along these lines, it was established that ubiquitination of Ca_v_3.2 T-type channels is an important mechanism for regulating pain signalling in primary afferent nociceptive neurons. For instance, Ca_v_3.2 channels are ubiquitinated within the III-IV linker of the channel by the ubiquitin ligase WWP1, whereas ubiquitin moieties can be removed by the ubiquitin protease USP5, hence controlling the ubiquitination/deubiquitination levels of the channel and its expression at the cell surface (Figure 5) [134]. In addition and consistent with the essential role of Ca_v_3.2 channels in pain signalling, *in vivo* knockdown of USP5 mediated analgesia in both inflammatory and neuropathic mouse models of mechanical hypersensitivity [134]. Importantly, analgesia was reproduced by *in vivo* disruption of Ca_v_3.2/USP5 interaction using interfering peptides or small organic molecules, revealing the potential therapeutic interest of targeting ubiquitination signalling as a new analgesic avenue [135,136].

Altogether, these data strongly support an important role of the UPS in the control of VGCCs expression. Although most attention has focused on the pore-forming Ca_v_α_1_ subunit, it is likely that ancillary subunits also undergo ubiquitination, which may provide an additional level of control over the expression of the Ca^2+^ channel complex.

### Asparagine-linked glycosylation

Asparagine (N)-linked glycosylation represents another post-translational modification recognized as essential for the expression and modulation of ion channels in general and VGCCs particularly [137]. N-glycosylation consists of the orchestrated enzymatic conjugation of an oligosaccharide tree (glycan) to an asparagine (N) residue in a consensus motif N-X-S/T (X being any residue although the nature of this residue strongly influences the probability of glycosylation) within the target protein. Although much attention has focused on the role of N-glycosylation in the folding and ER quality control of nascent proteins, the importance of N-glycosylation in the trafficking and stability of the channel at the plasma membrane has emerged. All of the ten pore-forming Ca_v_α_1_ subunits are glycosylated, and pharmacological or molecular disruption of canonical glycosylation sites has shed light on the importance of the glycan tree in the trafficking of the channel. For instance, disruption of the glycosylation of Ca_v_1.2 expressed in *Xenopus* oocytes caused a substantial decrease in the surface expression and Ca^2+^ conductance [138]. Biochemical evidence for glycosylation of the skeletal channel homologue Ca_v_1.1 is also available but no functional data have yet been reported [139].

**Figure 6:**
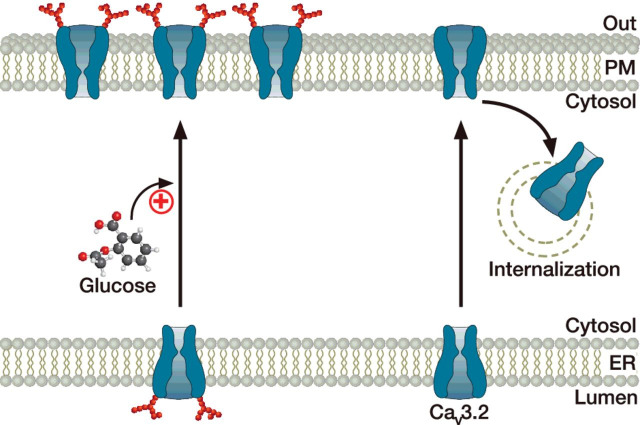
Glycosylation modulates surface expression of Ca_v_3.2 channels Although N-glycosylation of Ca_v_3.2, especially at asparagine residues N192 and N1466 (in the human channel), has little influence on the forward trafficking to the cell surface, it enhances the life-time of the channel in the plasma membrane by slowing down its internalization. In addition, N-glycosylation supports glucose-dependent potentiation of the trafficking of Ca_v_3.2 channels to the cell surface.

The most extensive characterization to date concerns T-type channels. The initial evidence for the glycosylation of T-type channels arose from biochemical studies of Ca_v_3.1 and Ca_v_3.3 channels. Although the apparent molecular weight of Ca_v_3.1 and Ca_v_3.3 varies with respect to their regional brain expression and also during neuronal development [140], enzymatic deglycosylation of the channels with PNGase F was sufficient to abolish these differences, suggesting that Ca_v_3.1 and Ca_v_3.3 do undergo glycosylation, but also that their degree of glycosylation differs with respect to their regional and developmental expression pattern [141]. However, it was only recently that the functional role of N-glycosylation in the trafficking of T-type channels was analysed. We and others have shown that glycosylation of Ca_v_3.2 channels at specific loci is essential for surface expression of the channel protein [142,143]. For instance, disruption of the glycosylation loci at asparagine N192 and N1466 in the human Ca_v_3.2 channel caused a significant reduction in the surface density of the channel that was not accompanied by a reduction in the total expression level of the channel, suggesting a role for N-glycosylation in the trafficking of the channel. Furthermore, analysis of the dynamics of the channels in the plasma membrane revealed that decreased surface expression of Ca_v_3.2 was largely caused by the enhanced internalization of glycosylation-deficient channels (Figure 6) [144]. In addition, we have reported that enhanced Ca_v_3.2 surface expression during chronic elevation of external glucose levels depends on the glycosylation of the channel [142,144]. This aspect may have important implications in chronic disorders such as peripheral painful diabetic neuropathy where enhanced expression of T-type channels is believed to contribute to the development and maintenance of this condition [145–148]. This notion is further supported by the observation that peripheral injection of neuraminidase in an animal model of diabetes reversed neuropathic pain [143]. Altogether, these data indicate that N-glycosylation of Ca_v_3.2 channels, in addition to influencing the gating properties of the channel [149], contributes to the maintenance of the channel protein in the plasma membrane, and may have important physiopathological implications.

In addition to the Ca_v_α_1_ subunit, glycosylation has been reported for α_2_δ and γ ancillary subunits [7]. As previously mentioned, the α domain of α_2_δ subunit is extensively glycosylated and initial reports suggested that N-glycosylation might stabilize its interaction with the channel [150]. In addition, it was proposed that glycosylation of α_2_δ is essential for supporting α_2_δ-dependent expression of Ca_v_1.3 and Ca_v_2.2 channels [151,152]. Recently, sequential disruption of a number of glycosylation sites in the α_2_δ-1 subunit, followed by examination of its surface expression, has uncovered a number of glycosylation loci that are essential for the expression of α_2_δ-1 at the cell surface [153]. However, it is not clear whether these effects are caused by a decreased trafficking of α_2_δ to the cell surface or a reduced stability of the protein in the plasma membrane. Consistent with the chaperone role of α_2_δ, Ca^2+^ currents in HEK-293 cells expressing Ca_v_1.2 channels were found reduced in the presence of glycosylation-deficient α_2_δ-1. It was, however, not established if this effect relied on the trafficking of the channel complex to the cell surface or instead involved the direct modulation of the channel activity. Indeed, a number of other glycosylation sites including asparagine N136 and N184 that initially were found essential for functional expression of Ca_v_2.2 channels [151], had little influence on the expression of α_2_δ-1 at the plasma membrane [153]. Interestingly, these glycosylation loci are located within the VWA-N region and may contribute to the functional interaction of α_2_δ-1 with the channel. This notion is supported by the observation that disruption of a glycosylation locus located in the VWA domain (N348) altered expression of Ca_v_1.2 channels [153]. Although a counterpart analysis of the surface expression of the Ca_v_α_1_ subunit in the presence of glycosylation-deficient α_2_δ-1 would be required to uncover the exact mechanisms by which glycosylation of α_2_δ influences expression of the channel, these data clearly indicate that N-glycosylation is an important factor contributing to surface expression of α_2_δ. It is worth noting that a number of glycosylation loci are located in the vicinity of the binding determinants of gabapentinoid drugs and may influence the pharmacology of the channel. Similar to α_2_δ, glycosylation of the γ-subunit seems to contribute to its expression at the plasma membrane, and disruption of the asparagine N48 located in the first extracellular loop connecting the first and second transmembrane segments disrupted the trafficking to the cell surface [154]. Interestingly, despite reduced surface expression of γ, Ca_v_2.2 currents were still inhibited, questioning the functional mechanism by which γ modulates the functioning of the channel [155].

## Mechanisms of subcellular targeting of VGCCs

Although the trafficking and dynamic of VGCCs at the plasma membrane are essential aspects, their specific targeting to subcellular loci is crucial for the maintenance of physiological regulation of normal neuronal activity. Although in some situations Ca^2+^ channels are widely distributed throughout the cell [156,157], they usually display a more restricted subcellular expression that allows them to fulfil specific functions. For instance, L-type channels are predominantly expressed in proximal dendrites where they are involved in dendritic Ca^2+^ signalling resulting from back-propagating action potentials and synaptic plasticity, and on cell bodies where they support activity-dependent modulation of gene transcription [158]. In contrast, N- and P/Q-type channels are primarily targeted to presynaptic terminals where they support voltage-dependent neurotransmitter release [159–162]. However, the detailed molecular mechanisms underlying the subcellular localization of VGCCs are still a matter of active investigations. Interaction of VGCCs with the β-subunit seems to contribute to the subcellular localization of the channel complex. Interaction of Ca_v_2.1 channels with the β_4_-subunit appears to contribute to the presynaptic targeting of the channel [163–165]. However, the detailed mechanisms of β-mediated targeting of the channel remain unclear. Interaction of Ca_v_2.1 and Ca_v_2.2 channels with a number of presynaptic proteins was also found to be important for the targeting of the channels into nerve terminals. This targeting relies on the binding of some of the proteins of the vesicular release machinery (SNAREs) including syntaxin 1A, SNAP-25 and synaptotagmin to the *synprint* region within the cytosolic II-III linker of the channel [166–168]. Consistent with this notion, Ca_v_2.2 splice variants that lack most of the *synprint* region fail to cluster into presynaptic terminals, and in contrast present an axonal expression profile [167,169]. The C-terminus of Ca_v_3.2 also contains a *synprint-like* region responsible for interaction with syntaxin 1A and SNAP-25 [170] and may contribute to the targeting of T-type channels to presynaptic terminals [171] where they may support activity-dependent low-threshold exocytosis [172,173]. Despite evidence for the role of channel/SNARE interactions in the targeting of the channels, transplanting the *synprint* region of Ca_v_2.2 into Ca_v_1.2 was not sufficient to trigger axonal expression or synaptic clustering of the chimaera channel, suggesting the involvement of other targeting mechanisms in the incorporation of Ca_v_2 channels into nerve terminals [167]. Along these lines, the C-terminus of Ca_v_2.2 contains a PDZ and SH3 binding motifs that are required for interaction with presynaptic adapter proteins Mint-1 and CASK, and contribute to but not essential for synaptic targeting of the channel [174,175].

The mechanisms responsible for somatodendritic targeting of L-type channels are less characterized but may involve interactions with structural proteins. For instance, Ca_v_1.3 channels associate with the postsynaptic adaptor protein Shank via a motif in the C-terminus of the channel, and co-localize at postsynaptic sites in hippocampal neurons [176]. Ca_v_1.3 channels are also found at presynaptic sites in sensory hair cells where they interact with Ribeye [177]. In photoreceptors, Ca_v_1.4 channels co-localize with the presynaptic scaffolding protein bassoon at the ribbon synapse [178]. A role for the β-subunit in the localization of L-type channel was also documented. For instance, point mutations in the AID region of Ca_v_1.2 that disrupt the interaction with the β-subunit abolished the dendritic clustering of the channel complex in hippocampal neurons [179]. Interestingly, although Ca_v_1.2 channels are targeted to the soma and dendrites of mature neurons, they are abundantly expressed in the growth cone of developing nerve cells [156]. It is conceivable that this switch in the expression pattern of Ca_v_1.2 switch might reflect the existence of a dynamic and reversible association of the channel with different β isoforms [180,181]. Consistent with this notion, a reshuffling of the molecular composition of the N-type channel complex during postnatal development was documented, from β_1b_ > β_3_ >> β_2_ at P2 to β_3_ > β_1b_=β_4_ at P14 and the adult stage [182]. Dynamic associations of β-subunits with Ca_v_1.1 channels also exist in skeletal muscles [183]. In addition, it was reported that assembly and disassembly of Ca_v_α_1_/α_2_δ-1a complex play an important modulatory role in the Ca^2+^ channel [184]. More recently, a polybasic plasma membrane binding motif consisting of a cluster of four arginine residues in the I-II linker of Ca_v_1.2 channels was identified. This motif forms a straight α-helix with the positive charges facing and interacting with negatively charged phospholipids in the plasma membrane. Besides modulating the gating of the channel, this cluster was found to be important for stabilizing the channel at the cell surface and neutralization of the arginine residues resulted in a decreased expression of the channel in the plasma membrane [185]. Considering the phospholipid asymmetry of the plasma membrane and related lipid-translocating enzymes, it is possible that this polybasic motif in Ca_v_1.2 contributes to the subcellular localization of the channel as was reported for the clustering of acetylcholine receptors [186].

## Concluding remarks and perspectives

The trafficking of VGCCs to the plasma membrane, and ultimately into subcellular loci, is an important aspect of the dynamics of these channels. To achieve this diversity, nerve cells have access to an extensive machinery from which they can select and assemble unique signalling systems with spatial and temporal properties necessary to control particular functions. In this review, we have highlighted some of the elements of this machinery underlying the expression of VGCCs, and it is likely that a number of additional players have not yet been identified. Whether some degree of redundancy exists as means of safeguarding critical neurological functions, it is clear that these systems are not perfect and the modified signalling pathways often results in the alteration of the expression of the calcium channels and consequently the emergence of multiple neurological disorders. However, the insights gained from understanding the fundamental mechanisms controlling the expression of these channels have opened new avenues for therapeutic intervention. The management of neuropathic pain with gabapentinoid drugs is one remarkable example, and recent results have emerged showing promising evidence that specific targeting of the trafficking of the calcium channel complex can be exploited as the basis for therapeutic intervention. This approach contrasts with the conventional and often unsuccessful method in targeting the channel activity *per se*, and undoubtedly opens new avenues for future drug development for a wide range of neurological disorders liked to calcium channel dysfunction [187].

## Abbreviations

AID: alpha-interaction domain
CaM: calmodulin
DRG: dorsal root ganglion
ER: endoplasmic reticulum
ERAD: endoplasmic reticulum-associated protein degradation
GK: guanylate kinase
HEK: human embryonic kidney
HVA: high-voltage-activated
KLHL1: Kelch-like 1
KO: knockout
LVA: low-voltage-activated
MIDAS: metal ion adhesion
NSF: N-ethylmaleimide-sensitive fusion protein
RRR: triple arginine
SNAP: soluble NSF attachment protein
SNARE: SNAP receptor
UPS: ubiquitin-proteasome system
USP: ubiquitin-specific protease
VGCC: voltage-gated calcium channel
VWA: Von Willebrand factor A
